# Metabolic changes in fibroblast-like synoviocytes in rheumatoid arthritis: state of the art review

**DOI:** 10.3389/fimmu.2024.1250884

**Published:** 2024-02-28

**Authors:** Zhipeng Hu, Yuan Li, Lili Zhang, Yayi Jiang, Caiyi Long, Qiyue Yang, Maoyi Yang

**Affiliations:** Hospital of Chengdu University of Traditional Chinese Medicine, Chengdu, Sichuan, China

**Keywords:** fibroblast-like synoviocytes, rheumatoid arthritis, metabolic changes, literature review, treatment implications

## Abstract

Fibroblast-like synoviocytes (FLS) are important components of the synovial membrane. They can contribute to joint damage through crosstalk with inflammatory cells and direct actions on tissue damage pathways in rheumatoid arthritis (RA). Recent evidence suggests that, compared with FLS in normal synovial tissue, FLS in RA synovial tissue exhibits significant differences in metabolism. Recent metabolomic studies have demonstrated that metabolic changes, including those in glucose, lipid, and amino acid metabolism, exist before synovitis onset. These changes may be a result of increased biosynthesis and energy requirements during the early phases of the disease. Activated T cells and some cytokines contribute to the conversion of FLS into cells with metabolic abnormalities and pro-inflammatory phenotypes. This conversion may be one of the potential mechanisms behind altered FLS metabolism. Targeting metabolism can inhibit FLS proliferation, providing relief to patients with RA. In this review, we aimed to summarize the evidence of metabolic changes in FLS in RA, analyze the mechanisms of these metabolic alterations, and assess their effect on RA phenotype. Finally, we aimed to summarize the advances and challenges faced in targeting FLS metabolism as a promising therapeutic strategy for RA in the future.

## 1 Introduction

Rheumatoid arthritis (RA) is a chronic autoimmune disease characterized by inflammatory arthritis as its main clinical manifestation and synovitis as the main pathological feature (1). Epidemiological data suggests that RA affects approximately 0.5–1% of the population, with some variations based on race and region (2). Despite an increased understanding of the disease, leading to reduced severity, disability, and mortality, RA prevalence continues to increase (3). Furthermore, as the understanding of RA pathogenesis deepens, fibroblast-like synoviocytes (FLS) are increasingly recognized for their important role in RA pathogenesis. The synovial membrane in a joint comprises a thin connective tissue structure consisting of an intimal lining layer and a sublining layer that covers the surface of most joints. The cells within the synovium consist mainly of two types: type A synoviocytes (macrophage-like synoviocytes) and type B synoviocytes (FLS), in addition to some other cells, such as macrophages and adipocytes (4).

RA pathogenesis can be divided into three stages: non-specific inflammatory, chronic inflammatory, and tissue damage (1, 5). In the non-specific inflammatory phase, individuals at high risk of RA develop non-specific inflammation of mucosal surfaces stimulated by various environmental factors. In the chronic inflammatory stage, synovitis development is a hallmark of RA onset. The pathology of this stage is characterized by synovial hyperplasia, neovascularization, and a heterogeneous inflammatory infiltrate, including lymphocytic pooling and germinal center-like structures. In the tissue injury stage, synoviocytes (especially fibroblasts) and proteases produced by the chondrocytes destroy the extracellular matrix of cartilage, ligaments, and tendons. FLS is a highly heterogeneous cell that plays different roles at different stages of RA. During the chronic inflammatory phase, FLS interacts with T cells, promoting T cell activation and helper T cell (Th)17 differentiation and producing inflammatory cytokines, chemokines, and matrix metalloproteinases, which are involved in the development of chronic inflammation in RA. During the tissue injury phase, cytokines such as interleukin (IL)-1, IL-6, and tumour necrosis factor alpha (TNF-α) directly promote FLS proliferation and activation, promoting cartilage and joint destruction (6). Furthermore, FLS may migrate through the bloodstream from inflamed joints, potentially “spreading” synovitis and promoting or exacerbating RA (7). However, the detailed changes in FLS at different stages of RA are still not fully elucidated.

Studies have found that daily resting calorie consumption is 8% higher in people with RA than in normal people. In addition, patients with RA may exhibit muscle wasting and body wasting due to increased catabolism (8). This metabolic abnormality in RA patients is closely related to the pathogenesis. FLS cell, as important component in the RA pathogenesis, play an important role in metabolic disorders in RA. During FLS activation, metabolic changes are considered a key factor in their functional alteration (9). Multiple lines of evidence indicate that RA FLS cells exhibit considerable differences in protein, glucose, and lipid metabolism than normal synovial FLS cells (6, 10–12). This abnormal metabolism is involved in RA pathogenesis, reflecting FLS adaptation to inflammatory and hypoxic conditions during excessive proliferation and phenotypic abnormalities. Targeting metabolism in FLS cells by inhibiting glycolysis reduces cytokine production and inhibits FLS proliferation, migration, and invasive phenotypes. Therefore, clarifying the metabolic difference between FLS cells in RA and normal synovium or in different stage of RA is beneficial to improve the understanding of the pathophysiology and treatment strategies of the disease.

In this review, we aim to summarize the metabolic differences between FLS in normal synovial tissue and those in RA synovial tissue. Understanding the metabolic characteristics of FLS and their role in RA pathogenesis will contribute to future drug development.

## 2 Abnormal changes in FLS activation and intracellular metabolism in RA inflammatory synovium

**Figure 1:**
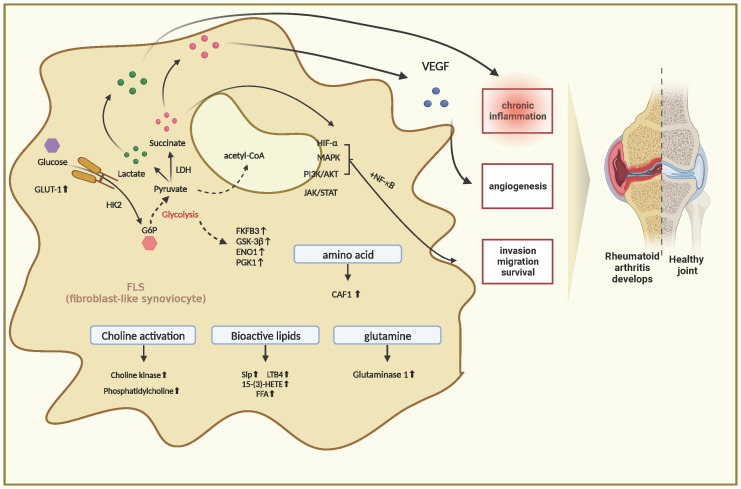
Metabolic Changes in Fibroblast-Like Synoviocytes in Rheumatoid Arthritis.

The metabolism of many substances within FLS cells is profoundly altered during RA pathogenesis. These complex metabolic processes are accompanied by the generation of different metabolites that induce intracellular signaling and effector functions. In a recent study, the authors analyzed the metabolite profiling of FLS in RA and compared it with that of FLS from osteoarthritis (OA) using gas chromatography/time-of-flight-mass spectrometry. The results revealed that compared with FLS in OA, there were 129 differential metabolites in FLS in RA, with 35 elevated and 26 decreased. These changes involved pathways related to glucose and amino acid metabolism. This study is important because it is the first to demonstrate important metabolic alterations in FLS cells in RA, providing new insights into the pathogenesis (13). These findings also confirm that FLS activation in RA is accompanied by multiple associated alterations in intracellular metabolic pathways, typified by alterations in gluconeogenesis, lipolysis, and amino acid metabolism. These alterations ultimately trigger inflammatory responses and synovial proliferation (**Figure 1**).

### 2.1 Abnormal glucose metabolism

In RA, FLS significantly increases in quantity and exhibits an abnormally aggressive phenotype. The rate of material metabolism of FLS cells increases to provide sufficient energy to the increasing quantity of FLS cells. An upregulation of glucose metabolism occurs in inflamed tissues of RA. The mechanisms may be associated with abnormalities in glucose metabolism intermediates and abnormal function of glucose metabolizing enzymes (14).

#### 2.1.1 Abnormal glucose metabolism intermediates

Increased glucose metabolism is a hallmark of cell proliferation and activation. In inflamed tissues, glucose metabolism shifts from oxidative phosphorylation to aerobic glycolysis. Glycolytic metabolism and intermediate metabolites of the mitochondrial tricarboxylic acid (TCA) cycle are involved in RA pathogenesis. Glycolytic metabolism is significantly increased in the inflamed synovial tissues of patients with RA, and inhibiting glycolysis reduces arthritic symptoms in animal models (9). The increased serum levels of lactate in patients with RA suggest an enhanced glycolytic metabolic process. Changes in energy metabolism within the joint result in decreased intracellular adenosine triphosphate, increased glycolysis, and higher lactate production. These metabolic changes can induce the invasion phenotype of RA synovial fibroblasts. In a study, the authors cultured FLS cells with lactic acid and found that the invasion and metastasis ability of FLS cells was significantly enhanced after 24 hours. The same results were also observed in experiments about succinic acid (15).

The glucose metabolism intermediates achieve this through several mechanisms.

First, they can increase the secretion of basic fibroblast growth factor by activating NF-κB through the monocarboxylate transporter, inducing the formation of angiogenic tubes. In addition, basic fibroblast growth factor can induce dysfunctional angiogenesis (a characteristic of inflammatory synovium), increase cell invasion, induce MMP13 synthesis in chondrocytes through the PI3K/Akt/ERK1/2 pathway, and activate the RhoGTPase protein, which is a key factor in promoting cell migration (Vegran F, Boidot R, Michiels C, et al. Lactate influx through the endothelial cell monocarboxylate transporter MCT1 supports an NF-κB/IL-8 pathway that drives tumor angiogenesis. Cancer Res 2011;1:2550–60.)(Im HJ, Muddasani P, Natarajan V, et al. Basic fibroblast growth factor stimulates matrix metalloproteinase-13 via the molecular cross-talk between the mitogen-activated protein kinases and protein kinase C delta pathways in human adult articular chondrocytes. J Biol Chem 2007;282:11110–21. 10.1074/jbc.M609040200)(Shin EY, Woo KN, Lee CS, et al. Basic fibroblast growth factor stimulates activation of Rac1 through a p85 betaPIX phosphorylation-dependent pathway. J Biol Chem 2004;279:1994–2004. 10.1074/jbc.M307330200).

Second, these metabolic changes can stabilize hypoxia-inducible factor (HIF)-1α in macrophages, inducing the synthesis and release of angiogenic growth factors, inflammatory cytokines, and extracellular cytokines, ultimately enhancing glycolytic activity (Colegio OR, Chu NQ, Szabo AL, et al. Functional polarization of tumour-associated macrophages by tumour-derived lactic acid. Nature 2014;513:559–63).

Altered FLS glucose metabolism induces an inflammatory response that stimulates enhanced cellular glucose metabolism, increased glucose transporter protein 1 (GLUT1) expression, and accelerated joint destruction (16). In the inflammatory state, the PI3K/AKT signaling pathway is activated and in turn causes downstream GSK-3β activation (17–19). GSK-3β can phosphorylate a range of downstream transcription factors, including mammalian target of rapamycin (mTOR). Activated mTOR can in turn have positive feedback on AKT protein, further promoting AKT activation and inducing the expression of key enzymes such as GLUT1, HK (20–22) In addition, it can also upregulate the expression of HIF-α (20–22). These aforementioned changes ultimately lead to a shift in cellular metabolism toward glycolysis. Correspondingly, the expression of important rate-limiting enzymes of glycolysis was increased (elaborated below).

GLUT1 is an important glucose transporter protein, and analysis of FLS samples from animal models and patients with RA has revealed elevated GLUT1 expression in inflamed tissues (23). Significant effects on various components of immune cells were observed in arthritis mouse models and FLS cells from patients with RA after GLUT1 knockdown. T-cell proliferation and B-cell antibody levels were markedly suppressed after GLUT1 knockdown. In addition to its effect on acquired immunity, GLUT1 also had a significant effect on innate immunity, with macrophage activity being significantly suppressed after GLUT1 knockdown (4). This result has been validated in animal models of other diseases, further confirming the important role of glucose metabolism in inflammation (23). The downregulation of glycolytic metabolism significantly inhibited FLS invasion and migration, angiogenesis, inflammatory mediator secretion, and the activation of HIF-1α, phosphorylation signaling, and transcriptional activation factor 3, including Notch-11C (24).

Significant difference in glucose (glycolysis and pentose phosphate pathways), acid metabolism (such as tyrosine-derived catecholamines and protein biosynthesis), and TCA cycle was observed in RA FLS compared with OA FLS (25). Results of studies on the metabolic profile of RA synovial tissue also confirm that the main changes in RA FLS are glucose and choline metabolism (26).

#### 2.1.2 Abnormal function of glucose metabolizing enzymes

PFKFB3 is a bifunctional enzyme that regulates the rate of glycolytic metabolism. Zou investigated the role of PFKFB3 in regulating FLS-mediated synovial inflammation and bone erosion and discovered that PFKFB3 expression was increased in FLS from RA compared with that from OA. Inhibiting PFKFB3 activity reduced cytokine and chemokine expression and decreased FLS proliferation, migration, and invasion (27). Inhibiting TNF-α-mediated activation of NF-κB and mitogen-activated protein kinase (MAPK) signaling reduces glucose uptake and lactate secretion. Intraperitoneal injection of PFKFB3 inhibitors in collagen-induced arthritis (CIA) rats reduces joint inflammation. Increased expression of PFKFB3 might promote the development of RA synovial inflammation and the invasive ability of FLS. Glycogen synthase kinase-3β (GSK-3β) is a serine/threonine protein kinase that regulates the inflammatory response. Kwon evaluated the inhibitory effect of selective GSK-3β inhibitors on FLS and discovered that GSK-3β inhibitors inhibited the production of FLS inflammatory mediators and reduced related gene expression in a dose-dependent manner. Administering GSK-3β inhibitors reduced the expression levels of NF-κB and phosphorylated amino-terminal kinase (JNK), c-jun, activating transcription factor (ATF-2), and p38. GSK-3β inhibitors reduced the severity of arthritic symptoms and histopathology, decreased serum levels of IL-1β, IL-6, TNF-α, and interferon-γ, and decreased the expression of T cells, macrophages, and interferon-γ in CIA rats. GSK-3β inhibitors significantly inhibited the inflammatory response to FLS in RA and CIA (28).

ENO1 is the rate-limiting enzyme in the glycolytic process. Fan revealed that, under hypoxic conditions, ENO1 gene expression was significantly upregulated in FLS of RA, leading to a corresponding increase in protein levels. This increased ENO1 expression significantly promoted the expression of anti-apoptotic protein Bcl-2 and cyclin B1, inhibited the expression of pro-apoptotic protein caspase 3, and promoted FLS proliferation (29). Applying small interfering ribonucleic acid (siRNA) transfection to inhibit ENO1 expression in FLS significantly reduced cell proliferation levels, suggesting that reduced ENO1 expression in FLS of RA could inhibit hypoxia-induced cell proliferation. Phosphoglycerate kinase 1 (PGK1) is a metabolic enzyme in glycolysis that catalyzes the conversion of 1,3-diphosphoglycerate to 3-phosphoglycerate, producing the first adenosine triphosphate in glycolysis.

Zhao discovered an enhancement in the expression of metabolic enzyme genes in the glycolytic process, such as ENO1, hexokinase (HK) 2, and PGK1, in the synovial tissues of CIA mice. Additionally, the expression levels of ENO1 and PGK1 genes and proteins were increased in the synovial tissues of patients with RA. High levels of PGK1 could be detected in the peripheral blood of patients with RA. *In vitro* experiments showed that following PGK1-siRNA transfection, the proliferation and migration ability of FLS was inhibited and the expression of IL-1β and interferon-γ in the culture supernatant significantly decreased. These results suggest that PGK1 is involved in the inflammatory process and synovial proliferation in RA (30).

HKs catalyze the first step of glucose metabolism, with HK2 constituting the major HK-inducible isoform. In one study, the authors first examined the expression of HK1 and HK2 in RA and explored the phenotypic changes in the migration and invasion of FLS cells following the knockdown or overexpression of HK2. The results indicated high expression of HK2 in RA tissues and low expression in OA. Overexpression of HK2 could promote the migratory and invasive abilities of FLS, aggravating RA (31).

### 2.2 Abnormal glutamine metabolism

Glutaminase 1 is the first catalytic enzyme of the glutaminolytic metabolic pathway. *In vitro* experiments have revealed that the differentiation of Th17 is dependent on glutaminolytic metabolism, leading to an upregulation of glutaminase 1 expression. Selective inhibition of glutaminase 1 activity, achieved through drugs or siRNA, inhibits Th17 cell differentiation and reduces the activity of mammalian target of rapamycin protein complex 1 (mTORC1) mediated by CD3/T-cell antigen recognition receptor (32). A study by Takahashi revealed that glutaminase 1 expression is increased in FLS in RA, and glutamine metabolism was increased (33). After glutamine was removed, FLS proliferation was decreased. Application of siRNA-transfected cells or administration of glutaminase 1 inhibitor inhibited FLS growth. Co-culture of IL-17 or PDGF with FLS increased glutaminase 1 expression levels. These results suggest that abnormal glutamine metabolism is involved in RA pathogenesis and glutaminase 1 regulates FLS proliferation (34).

### 2.3 Abnormal activation of choline kinase

Phosphatidylcholine metabolism affects cell proliferation, migration, and signaling and these mechanisms might be an important regulator of tumorigenesis. Choline metabolism is highly activated in FLS and may be a potential therapeutic target for RA.

Choline kinase is a phosphotransferase enzyme that catalyzes the conversion of choline to phosphocholine, the first step in phosphatidylcholine biosynthesis. Phosphocholine is a type of phospholipid and an important component of biofilms. Its molecular structure contains hydrophilic phosphate and oleophilic fatty acid groups, which can bind lipid substances with water and participate in emulsification. It is also an important component of brown fat in animal and plant tissues. The upregulation of choline metabolism, characterized by an increase in phosphocholine, is an important feature of tumor progression.

Choline kinase is highly expressed in RA synovial tissue and cultured FLS. TNF and PDGF stimulations increase choline kinase expression and phosphatidylcholine levels in FLS, suggesting the activation of this metabolic pathway in the inflammatory synovial microenvironment of RA (35). Choline kinase inhibitors (such as MN58b) inhibit the invasive capacity of FLS in RA *in vitro*, including the ability to migrate and resist apoptosis. In a model of arthritis with K/BxN seroconversion, pharmacological inhibition of choline kinase activity significantly reduced arthritic symptoms.

Another important enzyme is phospholipase D. Phospholipase D specifically degrades phosphatidylcholine, producing phosphatidic acid and choline. Phosphatidic acid is an important regulator of the inflammatory response, stimulating the secretion of inflammatory factors, including TNF-α, IL-1β, IL-6, nitric oxide, and prostaglandin E2, and mediating the activation of various cells. Choline kinase is an inflammatory factor that stimulates the transcription and secretion of the corresponding cellular factors, while the effects observed with choline kinase inhibition are at least partially related to phospholipids. This may be a potential mechanism by which phospholipase D participates in RA pathogenesis. Phospholipase D-specific siRNA or specific small molecule inhibitors significantly reduce FLS secretion of IL-6, IL-8, and CCL20 (36).

### 2.4 Bioactive lipid abnormalities

Sphingosine kinase (SphK) phosphorylates sphingosine to form sphingosine phosphate (S1P), an important bioactive lipid involved in the pathogenesis of many autoimmune diseases (37). S1P expression is upregulated in the synovial membrane and fluid of patients with RA, and S1P regulates the migration of osteoclast precursor cells and bone metabolic homeostasis, which are involved in RA bone destruction. SphK blockade inhibits cytokine and matrix metalloproteinase-9 secretion from peripheral blood mononuclear cells in RA. However, interfering with SphK2 in different models produced different effects. Using SphK2-deficient transgenic mice revealed that SphK2 deficiency had no effect on arthritis severity, whereas the use of a SphK2 inhibitor resulted in increased arthritis severity (38). Leukotriene B4 (LTB4) is an important inflammatory lipid mediator that mediates or exacerbates synovial inflammation in the K/BxN arthritis model, and TNF stimulates the production of high levels of leukotriene B4 in FLS. Animal studies have revealed that leukotriene B4 can promote the erosion of joints via vascular cataracts (39). The downstream product of 15-LOX, 15-S hydroxyeicosatetraenoic acid [15-(S)-HETE], a derivative of arachidonic acid, increased matrix metalloproteinase-2 protein and messenger RNA levels in the FLS of RA. Phosphatidylinositol 3-kinase inhibitors and NF-κB inhibitors (15⁃LOX) antagonized 15-(S)-HETE (40). This finding suggests that the altered cellular metabolism is associated with the activation of intracellular signaling pathways. Free fatty acids are basal metabolites that contribute to joint inflammation and destruction and stimulate FLS by binding to Toll-like receptor 4. Some bioactive lipids have anti-inflammatory effects, and phosphatidylserine inhibits IL-1β-mediated inflammatory responses in FLS and reduces arthritic symptoms in a keratin-mediated rat model (41).

### 2.5 Abnormal amino acid metabolism

CAT-1 is a major transporter protein of L-arginine and is overexpressed in RA FLS. CAT-1 is upregulated and promotes FLS proliferation through L-arginine uptake, promoting RA progression (37).

## 3 Altered FLS metabolism and intracellular signaling pathway transduction

Most of the stimulators mediating the activation of FLS activate specific receptors or channels located extracellularly or intracellularly and sequentially activate intracellular signaling proteins. MAPK and NF-κB are the most widely studied signaling pathways in FLS and are essential for FLS activation and differentiation into aggressive subtypes (42). Phosphatidylinositol 3-kinase signaling is mediated through AKT1 and mammalian target of rapamycin signaling proteins and downstream activation of the corresponding transcription factor HIF-1 (43). The Janus activating kinase/signal transducer and activator of the transcription signaling pathway is highly activated in inflammatory diseases such as RA. The p38 and p42/44 MAPK pathways, as well as the Rho kinase signaling pathway, regulate S1P-mediated cell migration and cytokine and chemokine secretion (44). Some signaling pathways can also inhibit the invasive ability of FLS. A study discovered that certain signaling pathways may inhibit the invasive ability of FLS (45). Under such conditions, these signaling pathways stimulate metabolic shifts within FLS cells to better accommodate and support changes in cell proliferation and phenotype through certain mechanisms. One of the most studied factors in this regard is hypoxia. HIF factors upregulate several glucose metabolism-related genes, including GLUT2, HK14, and lactate dehydrogenase, ultimately leading to a shift in cellular metabolism from oxidative phosphorylation to glycolysis (31, 46). Overall, the above signaling pathways increase the expression of glycolysis-related genes by upregulating the expression of HIF factors, shifting cellular metabolism to better support its function. However, the relationship between altered FLS metabolism and specific signaling pathways must be further investigated.4 FLS metabolic changes and functional abnormalities

Activation of FLS results in altered gene expression, production of new cytokines, chemokines, matrix-degrading enzymes, and increased cell proliferation, migration, and cartilage erosion. FLS is involved in synovial fluid formation, and increased synovial fluid secretion is an energy-consuming biosynthetic process (47).

### 3.1 Abnormal changes in synovial fluid composition and function in inflammatory joints with RA

In patients with RA, synovial fluid lubrication is reduced, and this functional change is associated with alterations in the composition of the synovial fluid. Lipidomic studies have revealed that the levels of lipids such as phosphatidylcholine, phosphatidylethanolamine, and sphingomyelin are higher in RA synovial fluid than in normal synovial fluid. Despite the increased levels of phospholipids in RA, the short lipid chains are ineffective in reducing friction during joint movement (48). Reduced lubricin levels in RA synovial fluid and decreased molecular mass of hyaluronic acid reduce the lubricating capacity of synovial fluid and transform it into an inflammatory signal via the Toll-like receptor 4/myeloid differentiation factor 88 pathway (49). RA synovial fluid contains higher lactate levels and reduced glucose content than normal synovial fluid, rendering the synovial fluid acidic. This phenomenon may be related to increased glucose uptake and enhanced glycolytic metabolism in the synovial membrane. FLS synthesizes large amounts of extracellular matrix and synovial glycoproteins to maintain joint function. Further research is needed on how glucose or other nutrients and metabolic changes affect FLS glycoprotein biosynthesis and synovial fluid properties.

### 3.2 Abnormal angiogenesis in RA inflammatory synovium

FLS proliferation causes hyperproliferation of synovial tissue and increases oxygen consumption in the synovium, creating a hypoxic microenvironment. This hypoxia is the main cause of neovascularization in RA synovial tissues. Hypoxia contributes to HIF activation, mediating the expression of related genes, including vascular endothelial growth factor, promoting synovial neovascularization, and contributing to the continued progression of RA (50). FLS and activated synovial macrophages secrete vascular endothelial growth factor, angiopoietin-2, placental growth factor, and fibroblast growth factor, which play important roles in neovascularization. Hypoxia activates glycolytic metabolism, and enzymes required for glycolytic metabolism (glucosyl-6-phosphate isomerase) and intermediate products of glycolytic metabolism (lactate and succinate) are secreted extracellularly, potentially acting as stimulators of neovascularization and facilitating the progression of angiogenesis.

### 3.3 Altered adhesion, migration, and erosion ability of FLS cells

FLS hyperproliferates in the synovium of RA and has a migratory, erosive, and cartilage-destroying phenotype. Synovial tissue from patients with RA has higher lactate levels than uninflamed synovial tissue, a consequence of increased levels of glycolysis in the synovial tissue under anaerobic conditions. This alteration results from cellular adaptation to anaerobic conditions, which allows FLS cells to better utilize energy under hypoxic conditions (51–53).

#### 3.3.1 Cellular metabolism influences the abnormal phenotype of FLS through multiple pathways

First, under hypoxic conditions, HIF-1α expression is increased, and downstream glucose metabolism-related genes such as GLUT2, HK14, and LDH are upregulated in the RA FLS, leading to increased glycolysis levels. This adaptation to inflammatory and hypoxic conditions has profound implications for FLS cell survival and function, with FLS cells with higher levels of glycolysis having greater proliferation and invasive capacity. In a mouse model of RA, glucose metabolism was increased in stromal cells, and glycolytic inhibition impaired FLS function and eliminated joint inflammation and damage (9, 54).

The second way in which metabolism influences the FLS phenotype is through the integrin pathway. Multiple integrins, especially β1-family integrins, are overexpressed in FLS with RA and affect the survival, proliferation, adhesion, and migration activities of FLS cells (55). Multiple metabolism-related signaling proteins, including AMPK, Mtorc1, and HIF-1, modulate the aberrant phenotype of FLS cells by regulating the expression and function of integrins (56).

Metabolism-related intermediates can act as inflammatory mediators driving chronic inflammation. For example, succinate, a TCA cycle intermediate produced in RA synovial fluid, enhances the release of IL-1β from macrophages through the succinate receptor Sucnr1/GPR91 mechanism (25). Additionally, succinate contributes to angiogenesis through HIF-1α and increases the expression of vascular endothelial growth factor through the GPR91 receptor (57).

### 3.4 FLS proliferation and apoptosis abnormalities

Apoptosis is a mechanism by which cells regulate their proliferation or respond to deoxyribonucleic acid damage through programmed death. The most distinctive feature of FLS in RA, apoptosis resistance, has altered mitochondrial pathways related to apoptosis and can resist receptor-mediated apoptosis at multiple levels, including dysfunction of Bcl2 family proteins, dysregulation of NF-κB signaling, p53 mutations, and low expression of p53 upregulated apoptosis regulator (58). Other mechanisms include the translocation of important metabolic enzymes involved in glycolysis to the nucleus, exerting anti-apoptotic effects, and potentially linking metabolic processes and apoptosis. HK2, which catalyzes the phosphorylation of glucose to form glucose 6-phosphate, binds to the mitochondrial membrane surface by interacting with the extracellular portion of voltage-dependent ion channel proteins (59). The interaction between HK2 and voltage-dependent ion channel inhibits the release of pro-apoptotic proteins, protecting cells from apoptosis. The increased expression of HK2 in FLS in patients with RA compared with patients with OA may play an important role in FLS apoptosis resistance in RA. FLS in RA has a higher level of glycolytic metabolic processes and increased anaerobic respiration and lactate production, creating an acidic extracellular matrix that protects FLS from capsaicin-mediated apoptosis by regulating calcium activity.

Autophagy, a biological process for intracellular organelle renewal and maintaining intracellular homeostasis, is essential to determine cell survival. Mitochondrial autophagy, responsible for removing dysfunctional mitochondria, is essential for maintaining normal metabolism and cell survival signals (60). The expression of autophagy-related proteins beclin-1 and LC3 is increased in FLS of RA, and autophagy gene expression inversely correlates with apoptosis (61). RA FLS increases autophagy through the endoplasmic reticulum stress response, resisting apoptosis. The relationship between mitochondria, apoptosis, and autophagy in FLS must be further investigated.

MiR-126 overexpression decreased PIK3R2 protein, promoted proliferation, and decreased RA-FLS apoptosis, whereas miR-126 inhibition increased apoptosis. Additionally, miR-361-5p promoted FLS proliferation and inhibited apoptosis by targeting ZBTB10 in RA, and its possible mechanism of action involved increasing RA-FLS cells that secrete inflammatory factors (62).

In hypoxic environments, RA-FLS cells exhibited a more pronounced increase in the expression of HIF-1α transcriptional regulatory genes, higher BNIP3 expression, and stronger mitochondrial autophagy and proliferative activity (63).

### 3.5 Interactions between synovial cells and immune cells

In the past, FLS was considered to be a passive recipient of inflammatory cytokine stimulation in joints, but in recent years, this view has been challenged with the gradual deepening of research. Research has found that FLS can interact with various immune cells, playing an important role in recruiting immune cells and slowing inflammation.

Interactions between multiple cells in synovial tissue determine the pathological changes in synovial tissue and RA development, with different cells interacting in two main ways: secretion of inflammatory mediators and direct intercellular contact mediated by receptors and ligands. Metabolic alterations contribute to the exchange of metabolites between different cells, influencing the development of chronic inflammation in RA. Intermediates of glucose metabolism and the mitochondrial TCA cycle have functions such as promoting neovascularization, cellular invasion, and anti-apoptosis. Branched-chain amino acids can influence cellular signaling, mediating mammalian target of rapamycin pathway activation (64). Lipid signaling is achieved through the activation of various receptors, including G protein-coupled and nuclear receptors. Moreover, several different types of lipid membranes have been identified to function as signaling molecules and intracellular messengers (65). Further research is needed to explore the specific types of lipid membranes functioning as signaling molecules and intracellular messengers, as well as the specific mechanisms of interaction between FLS and other synovial cells.

### 3.6 Epigenetic alterations

Large body of evidence suggesting that epigenetic regulation has an important impact on FLS proliferation and phenotype. Changes in the epigenetic landscape of genes associated with nutrient transport proteins reveal a potential role of the SLC family in FLS metabolism. The SLC family is involved in glutamate transport, basal glucose uptake, bicarbonate transport, amino acid transport, monocarboxylate transport, zinc and other small amounts of metal transport, and cellular exocytosis of iron. These activities promote the activation of FLS in multiple ways, whereas the use of related metabolic process inhibitors can reduce FLS migration, reducing bone erosion and joint edema. Moreover, they can be used as innovative targets for treating RA by targeting and regulating the activation of SLC family members during the development of new drugs (66).

Despite these advancement, current research on epigenetic regulation of FLS still has major limitations, which are mainly manifested in several aspects.

Firstly, for the human genome, the number of LncRNAs is extremely large, far exceeding that of coding RNAs, which brings a huge workload to research (67–69).

Secondly, the numerous LncRNAs do not act alone, they interact with other components to form a complex network of interactions. However, current research on LncRNA has mostly focused on a single LncRNA, studying its function through overexpression or silencing, without deeply revealing the interactions between a certain LncRNA and other LncRNAs, DNA, coding RNA, and proteins in this complex network of interactions (69).

More importantly, it is still unclear what the key regulatory targets are in this pathogenesis network.

In addition, the latest research has also found that lncRNAs in tissues have tissue specificity. LncRNAs have different expression characteristics and functions in different tissues, which poses new challenges for studying the function of LncRNAs and regulating FLS (70). The above analysis indicates that the epigenetic regulation of FLS, although seemingly a very attractive field, still has a lot of unknowns to explore, and there is still a long way to go from preclinical research to clinical application (71).

### 3.7 Osteogenic differentiation

FLS can differentiate into osteoblasts; however, the driving factor and the underlying mechanism remain unclear. In a recent study, the authors conducted micro-RNA array analysis to identify differentially expressed micro RNAs and investigated their role in driving FLS differentiation. The results revealed that miR-218 is a key factor in FLS differentiation into osteoblasts. The mechanism may be associated with the targeting of the ROBO1/DKK-1 axis. This study introduces a new therapeutic strategy to promote FLS differentiation into osteoblasts by increasing the miR-218 reserve and attenuating structural damage (72).

### 3.8 Difference in metabolic changes between FLS in different disease stage of RA

Much of the research to date on the metabolic changes that occur in FLS during the pathogenesis of RA has focused on RA compared to osteoarthritis, or RA compared to normal synovial tissue. However, such studies do not allow us to know whether these metabolic alterations occurring in FLS are present before RA pathogenesis occurs, i.e., contributing to RA pathogenesis, or whether they are simply a response of FLS cells to RA pathogenesis. As mentioned earlier, the pathogenesis of RA is a gradual progression in which FLS plays different roles in different stages, and the study of alterations in FLS metabolism in different stages of RA will help us to gain a deeper understanding of the RA pathogenesis. Evidence now suggests that metabolism FLS is significantly lower in basal mitochondrial respiration, ATP production, and maximal mitochondrial respiration in RA compared with normal tissue, whereas there were no significant differences in the RA risk group compared with controls (9, 60, 73). This suggests that the differences in mitochondrial respiration between RA and normal tissues may be the result of disease-induced effects rather than the cause of disease onset, as these abnormalities are present as early as the onset of RA.

Metabolic flexibility indicates the ability of cells to adapt to changing environments (74). In terms of metabolic flexibility, one study examined differences in the metabolic flexibility of FLS when one of the three pathways, glucose, fatty acids and glutamine, was inhibited. The results found that FLS in the control group had better metabolic flexibility, whereas FLS in the RA and RA risk groups relied primarily on fatty acid oxidation and had poorer metabolic flexibility (73). Considering that metabolic flexibility has emerged as one of the potential targets for aging-related diseases, this result may be of value for future drug development.

Analysis of differences in lipid metabolism in FLS cells showed similar levels of lipid metabolism in RA and RA-risk populations, both of which were significantly lower than normal FLS cells (75, 76). These results suggest that this lipid metabolism abnormality may be one of the factors driving disease progression, as it precedes the onset of arthritis. Further studies found impaired mitochondrial β-oxidation in FLS in RA and RA risk groups compared to normal FLS (73). However, what causes the impaired mitochondrial β-oxidation capacity is still not well understood. These results suggest that FLS cell metabolism is a very complex issue and still needs to be further studied and elucidated.

### 3.9 Implications for development of metabolic targeted therapies for RA

#### 3.9.1 Targeted bone metabolism for RA

##### 3.9.1.1 Interleukin-1 receptor-activated kinase 4 inhibitors

TLR is a pattern recognition receptor that plays an important role in the activation of innate immunity (77). Activation of FLS cells in RA is mediated by the Toll-like receptor TLR signaling pathway. Interleukin-1 receptor-activated kinase 4 (IRAK4) is a core regulatory modulator of the innate immune response. IRAK4 activates NF-κB, interferon regulatory factor 5 (IRF-5), and MAPK by binding to the adaptor protein myeloid differentiation factor 88 (MyD88) to transmit signals from IL-1R and TLRs (78–80). In RA, IRAK4 inhibitors attenuated RA disease activity by blocking TLR7-induced M1 or FLS activation and Th1/Th17 cell polarization. In addition, IRAK4 inhibitors disrupted RA osteoclastogenesis thereby attenuating bone erosion, which makes targeting IRAK4 an attractive therapeutic target (81, 82).

Lee KL et al. developed a selective and potent IRAK4 inhibitor, PF-06650833, and demonstrated its good efficacy in preclinical and clinical studies in a study (83). In an article, the author conducted two placebo-controlled phase 1 clinical studies with a duration of 96 and 14 days, respectively, to explore the safety, tolerability, pharmacokinetics, and pharmacodynamic characteristics of PF-06650833 in single and multiple incremental dose trials in healthy subjects. The results showed that PF-06650833 exhibited good safety and tolerability in both studies (84). In another study, the author further explored the therapeutic effect of PF-06650833 on RA. Studies using cell models have shown that PF-06650833 significantly reduces the release of cytokines and matrix metalloproteinases (MMPs) in response to all ligands. However, it did not have a significant effect on the release of cytokines in IL-1 induced FLS. Using the rat collagen fiber induced arthritis model (CIA model), PF-06650833 was found to significantly inhibit *in vivo* inflammation in rats, similar to tofacitinib. Subsequently, the author explored the effect of PF-06650833 300mg/d (n=7) or placebo (n=14) on the inflammatory marker IFN signature in healthy subjects after 14 days of treatment. The results showed that compared to before treatment, the IFN signature of the placebo group increased by 9.1%, while the IFN signature of the experimental group subjects decreased by 28.8% (85).

Based on this result, Pfizer has conducted two additional Phase II clinical studies (NCT02996500, NCT04413617). NCT02996500 is a phase 2, multicenter, randomized, double blind, double simulated, placebo, and active controlled clinical study that evaluated the efficacy and safety of PF-06650833 in patients with moderate to severe active RA who had poor response to methotrexate after 12 weeks of treatment. NCT04413617 is a 24-hour multicenter, positive controlled RCT study aimed at evaluating the efficacy and safety of pf-06650833, pf-06651600 (ritalcitinib), and tofacitinib alone and in combination in patients with moderate to severe active rheumatoid arthritis with insufficient methotrexate response. At present, both studies have been completed and the relevant papers have not been published.

#### 3.9.2 CDK4/6

Abnormal proliferation of FLS cells can directly cause cartilage and bone damage, so how to reduce this abnormal phenotype has become one of the research directions. In recent years, some research on the mechanisms of bone erosion caused by FLS has helped to discover new therapeutic targets. Cyclin dependent kinase inhibitor (CDKI) is an important protein that inhibits cell cycle and can inhibit rheumatoid inflammation by downregulating the expression of type I interleukin-1 receptor (IL-1RI) and inhibiting JNK activity. Research has shown that inhibiting the CDK4/6 activity of FLS can inhibit the production of various inflammatory mediators, including IL-1 and MMP-3 (86).

AP-1 is one of the most important transcription factors in inducing genes downstream of TNF-α in FLS. T-5224 is an AP-1 targeted inhibitor that has achieved good results in RA animal studies. However, no results were reported in Phase II clinical trials (87).

A recent study showed that inhibiting CDK4/6 not only inhibits the proliferation of synovial fibroblasts, but also directly exerts a cartilage protective effect, which is mainly achieved by inhibiting the stability of JUN and weakening the transcription activity of AP-1 (88).

#### 3.9.3 Targeting HIF

HIF is a core transcription factor that cells adapt to hypoxic conditions and cause corresponding metabolic changes. It can transcribe and activate downstream genes that regulate oxygen balance and metabolic activation, thus playing an important role in many diseases. In RA, an increase in hypoxia can lead to an increase in the degree of synovitis, mainly achieved by promoting tissue inflammation and FLS invasion phenotype (89, 90). In addition, hypoxia may also lead to an increase in leptin expression in FLS, leading to reduced osteogenesis and increased adipogenesis (91). HIF-1 α Under hypoxic conditions, it can directly regulate the expression of Notch-1 and Notch-3 genes, thereby promoting the invasion, metastasis, and angiogenesis of FLS.

In a study, the authors used N1ICD and N3ICD inhibitor LY411575 to intervene in collagen induced arthritis model rats. The results showed that the disease symptoms and severity of arthritis in the model rats were significantly improved after intervention (92).

#### 3.9.4 Glucose metabolism

Among the key enzymes involved in glucose hydrolysis, three enzymes have been considered to have potential therapeutic value. The first type is bifunctional PFKFB3 enzyme. In FLS, the use of PFKFB3 small molecule inhibitors to intervene in FLS reduces glucose uptake, leading to a decrease in lactate production (15, 27). The second rate-limiting enzyme with potential therapeutic value is hexokinase 2 (HK2), which is an attractive target because it has little impact on T cell-mediated immune responses, meaning that targeting HK2 will not have a significant impact on the systemic immune response (93). In a study, the authors injected HK2 inhibitor, 3-bromopyruvate (BrPA), into mice with arthritis models, which inhibited glycolysis levels and resulted in a significant decrease in arthritis levels (9). In another study, researchers used another HK2 inhibitor, 2-deoxyglucose, which also achieved good results (24). The last one is phosphoglycerate kinase (PGK)1. Targeted knockout of PGK1 in cell models reduces inflammation levels, cell proliferation, and cell migration activity (30).

#### 3.9.5 Intermediate products of glucose metabolism

Cyclin dependent kinase (CDK) is a group of serine/threonine protein kinases. CDK drives the cell cycle through chemical interactions with serine/threonine proteins, and works synergistically with cyclin, making it an important protein in cell cycle regulation. Seliciclib is an oral cyclin dependent kinase (CDK) inhibitor originally developed for the treatment of tumors. Data from animal experiments have shown that CDK inhibitor R-roscovitine (Seliclib or CYC202) can inhibit disease activity in arthritis mice and promote FLS cell apoptosis (94). The TRAFIC study is a two-part, multicenter, phase 1b/2a clinical study involving a total of 15 participants, aimed at exploring the safety and tolerability of Seliclib in RA patients receiving biologic therapy. This study is the first clinical study aimed at targeting FLS proliferation and apoptosis activity. It has determined the maximum tolerable dose of seliclib and confirmed its good safety and tolerability, laying the foundation for further large-scale clinical research in the next step (95).

#### 3.9.6 Lipid metabolism and related downstream pathways

##### 3.9.6.1 Ferroptosis

ferroptosis is a programmed cell death, mainly caused by the accumulation of lipid peroxides in cells. Initially, ferroptosis was considered a potential target for cancer treatment, but recent research suggests that ferritin may be related to immunity and play a role in affecting tissue inflammation, cell growth, and apoptosis (96). In a study, it was found that TNF can inhibit the occurrence of ferroptosis by upregulating multiple proteases, including solid carrier family 7 member 11 SCL7A11. The combined intervention of TNF antagonists and iron death inducers can effectively induce iron death in FLS cells, thereby significantly weakening the disease progression of collagen induced arthritis models (97). In addition, it was also found in the study that the bioactive peptide Galectin-1 Derived Peptide 3 G1dP3 promotes ferroptosis in FLS cells through the p53/SLC7A11 axis (98).

However, ferroptosis in FLS is still an emerging field, and there is still limited research on targeted drugs to improve ferroptosis in FLS for the treatment of RA. Although drugs, including JAK inhibitors, have been found in studies to improve ferroptosis, they lack specificity and are not specifically targeted for ferroptosis.

Main targets and intervention of metabolic therapies for RA is summarized in **Table 1**.

**Table 1:** Main targets and intervention of metabolic therapies for RA.

	Target	Intervention	Preclinical or clinical	Comments	Reference(s)
	IRAK4	IRAK4 inhibitor, PF-06650833	Preclinical and clinical	Exhibited good safety and tolerability in both studies	84, 85
			NCT02996500,NCT04413617	both studies have been completed and the relevant papers have not been published	–
	Cyclin dependent kinase inhibitor	CDK4/6	Preclinical	not only inhibits the proliferation of synovial fibroblasts, but also directly exerts a cartilage protective effect	88
	Notch	N1ICD and N3ICD inhibitor LY411575	Preclinical	The disease symptoms and severity of arthritis in the model rats were significantly improved	92
Glucose metabolism	bifunctional PFKFB3 enzyme	PFKFB3 small molecule inhibitors	Preclinical	Leading to a decrease in lactate production	15, 27)
	Hexokinase 2 (HK2)	–	Preclinical	it has little impact on T cell-mediated immune responses, meaning that targeting HK2 will not have a significant impact on the systemic immune response	93
	Hexokinase 2 (HK2)	HK2 inhibitor, 3-bromopyruvate (BrPA)	Preclinical	Inhibited glycolysis levels and resulted in a significant decrease in arthritis levels	9
	Hexokinase 2 (HK2)	2-deoxyglucose	Preclinical	Achieved significant improvement	24
	phosphoglycerate kinase (PGK)1	–	Preclinical	Targeted knockout of PGK1 in cell models reduces inflammation levels, cell proliferation, and cell migration activity	(30)
Intermediate products of glucose metabolism	Cyclin dependent kinase (CDK)	oral CDK inhibitor, Seliciclib	Preclinical	Data from animal experiments have shown that CDK inhibitor R-roscovitine (Seliclib or CYC202) can inhibit disease activity in arthritis mice and promote FLS cell apoptosis	94
	CDK	Seliclib	Clinical	This study is the first clinical study aimed at targeting FLS proliferation and apoptosis activity. It has determined the maximum tolerable dose of seliclib and confirmed its good safety and tolerability, laying the foundation for further large-scale clinical research in the next step	95

## 4 Concluding remarks

The altered intracellular metabolism of FLS is due to stress responses in the microenvironment of inflamed tissues. In inflamed tissues, major nutrients, including sugar, glutamine, and oxygen, trigger new metabolic manifestations. The altered metabolism increases the exchange of metabolic substances between fibroblasts and other types of synovial cells, potentially activating FLS. This activation contributes to the initiation of an immune response or abnormal immune response processes, leading to joint bone invasion and destruction and inducing or accelerating RA.

Based on this understanding, numerous studies have been conducted to unravel FLS metabolism, with a focus on exploring the possibility of targeting FLS substance metabolism for RA treatment. However, owing to the complexity of the disease, many questions remain to be understood about the relationship between FLS and RA. Further exploration of this issue is imperative in the future.

## Author contributions

ZH: conceptualization and writing – review & editing. MY: literature collection and writing – original draft. QY, YL, YJ, and CL: literature collection. LZ: Writing – original draft.
